# Factors affecting desired participation in transition to an adult life with Duchenne muscular dystrophy (DMD)

**DOI:** 10.1177/22143602251324847

**Published:** 2025-03-03

**Authors:** Laura JB Merkenhof, Yvonne Veenhuizen, Elizabeth Vroom, Greet Sterenberg, Wendy CHM Hesseling, Jan T Groothuis, Edith H Cup, Saskia LS Houwen- van Opstal

**Affiliations:** 1 Department of Rehabilitation, Donders Institute for Brain, Cognition and Behaviour, Radboud University Medical Center, Amalia Children's Hospital, Nijmegen, The Netherlands; 2 Department of Rehabilitation, Donders Institute for Brain, Cognition and Behaviour, Radboud University Nijmegen Medical Centre, Nijmegen, The Netherlands; 3 Duchenne Parent Project, Veenendaal, the Netherlands; 4 We Jane, Amsterdam, The Netherlands

**Keywords:** adult, transition, Duchenne muscular dystrophy, participation, social domain

## Abstract

**Background:**

For people with Duchenne muscular dystrophy (DMD), the transition into their desired adulthood can be challenging.

**Objectives:**

This study aims to; (1) exploring the desired participation for (young) adults with Duchenne muscular dystrophy (DMD); (2) exploration of the view and role of parents in this process; and (3) capturing the gap and the influencing factors between the current and desired situation.

**Methods:**

A cross-sectional digital survey was conducted, based on literature, expert opinion, and interviews with young adults with DMD and their parents. Descriptive and non-parametric statistics were used.

**Results:**

43 adults* with DMD an 30 parents completed the survey. All adults with DMD wanted to have an independent life. They were satisfied with their leisure activities. Gaps between the desired and current situation were identified concerning facilities and aids, social activities, and employment. Important factors of influence on these topics were accessibility, outdoor mobility, adequate care facilities, self-confidence, adequate knowledge of professionals and caregivers, and support of parents. The 30 participating parents saw limited opportunities in accessing facilities and aids, job opportunities, and their son having an intimate relationship.

**Conclusions:**

Adults with DMD desire a meaningful and independent life. The challenges they, and their parents face are mainly on social participation. More focus and collaboration is needed between health care services, social services and occupation environments to empower people with DMD living their desired adult life.

*Adults; In this article, we use the term adults. By this we include boys aged 16 and 17.

## Introduction

Transition and becoming independent in participation takes place in our everyday lives and becomes more important with age. For example starting school, puberty, developing relationships, seeking employment. A major transition period is adolescence, in which decisions about lifestyle, education and vocation become important.^1^ Most adolescents experience multiple transitions during this unstable developmental period, including changes in daily activities and roles, health care, and relationships (e.g., family, friends, students, colleagues, and intimate others). The developmental period from the teenage years through the twenties is a time of profound transition to increased independence and adult role attainment.^2^

For people with Duchenne muscular dystrophy (DMD), the transition into adulthood can be even more challenging. DMD is a progressive neuromuscular condition with a decline in skeletal muscle function, cardiac and respiratory function. Due to the current standards of care, including cardioprotective management, the use of corticosteroids, scoliosis correction, and ventilatory support, the life expectancy has increased from the early twenties until in their late thirties, and DMD is no longer a childhood condition.^3–5^ At ages when adolescents generally have a desire for greater independence, those with DMD often have increasing healthcare needs and physical reliance on others for activities of daily living, which can create challenges in making a successful transition to adult lifestyles.^6^ Besides, parents experience that providing care for their adult son with DMD is increasingly burdening as the disease as well as the age of the parents progresses.^7^

Previous research on transition into adulthood showed that a majority of adolescents with DMD had limited postsecondary education, were unemployed, single, and continued to live with their parents.^8–10^ Additionally, a survey on participation domains in adolescents and adults with DMD showed that restrictions were experienced in leisure activities, contact with others, mobility, going out, and visits from or to friends and family.^11^ Research on meaningful occupation within a population having neuromuscular disorders showed that a lack of support and resources, negative attitudes of others, and lack of motivation in pursuing meaningful occupations were important barriers.^12^ Qualitative research on adolescents with DMD, their parents, and clinicians showed that relational factors (i.e., effective communication and family involvement), leadership and advocacy were reported as enablers of transition, while the (dis)continuity and (un)availability of care could be a barrier and an enabler.^13^ The same study reported mental health concerns as a challenge for adults with DMD.

Despite this knowledge, the transition into adulthood as well as participation in adult life are often insufficiently addressed within the growing population of adults with DMD. Experience from clinical practice shows that together with their parents, adolescents with DMD often gradually enter a situation in which care takes a larger part of their time and their social participation decreases. On the other hand, assumptions about a successful transition can be normative and do not always align with the desired future perspective of adults with DMD.^14,15^

In this study, we focus on the perspective on participation and daily occupation of young adults with DMD and their parents. We aim to explore the desired situation for (young) adults with DMD and their parents, their current situation, the care giver burden, and factors which can diminish the gap between the desired and current situation.

## Materials and methods

### Design and participants

A cross sectional survey was set out among the Dutch population with DMD, aged 16 years and older and their parents. The adults with DMD and/ or their parents were approached per email by the Duchenne Parent Project (DPP) (n = 95), and through social media (Instagram) of DPP. Participants (had to) have (a son with) DMD, and an age above 16 years. Informed consent was obtained from all participants and/or from their parents. Participants received a 10 euro gift card after completing the survey.

### Survey

#### Development of the surveys

Two surveys were used in this study; one for the (young) adults with DMD, and one for their parents. Both surveys can be found in the Supplementary Material. The surveys could be filled out digitally, sent a paper version by mail, or via telephone call.

The surveys were compiled using five steps:

1. A quick review of the current literature in Pubmed.
2. An expert meeting which was organized to have consensus on the topic list of the following interviews. The expert meeting consisted of the following participants:
  Three care professionals from the neuromuscular center of the Radboudumc (two occupational therapists and one rehabilitation physician), who have a large experience in clinical care for adolescents and adults with DMD.
  Experience expert: an adult with DMD.
  Two external care givers, who have experience as care givers for adults with DMD.
  Two delegates from WeJane, a qualitative research agency with experience in the DMD population.
  Two delegates from DPP, patient advocacy of DMD in the Netherlands.
  Two delegates from Emile Thuiszorg, a home care organization.
3. Interviews with 14 adults with DMD and their parents. The interviews were held by the two delegates of WeJane at people's homes for 3–4 h. In this time frame the adults were interviewed together with their parents, but also separately. If this was of additional value, the delegates joined them to their school or sports. Videos of these visits were analyzed by the researchers of WeJane and the care professionals of the Radboudumc. For details on this pilot phase, see report 1 in the Supplementary Material. The common tread in the interviews were the differences in the desired and actual situation and the parent role.

Topics with a great influence on the desired autonomy and desired participation in the perspective of adolescents and adults with DMD and their parents were collected and categorized. The topics were converted in statements and questions for the survey.

1. A second expert meeting with the same delegates as described under 2) was used to discuss and refine the survey.
2. The survey was tested and refined with help of an adult with DMD who was not involved in the development of the survey to test language use, feasibility, and comprehensibility.

The surveys for the young adults and their parents are found in the second part of the Supplementary Material.

#### Structure of the surveys

The survey consisted of a general part and specific parts per topic. The general part consisted of patient characteristics, the amount of care, and 20 statements. Those statements were mainly on live goals and relationships with the parents as this were important issues in the interviews. Participants could indicate on a 5 point Likert scale to what extent they agreed (range 0 (does not apply to me at all) to 5 (does very apply to me). The topic specific part consisted of questions on experienced differences between the desired situation and the current situation for the participant. Besides, we included open fields to reflect on decisions and to elaborate on advices they would give their younger peers. The survey for parents included questions about the general characteristics of the parents, general characteristics of their son, the current and desired situation about their family, the Zarit burden interview (ZBI), and scores on statements (see Supplementary Material, part 2). The ZBI is a validated questionnaire, which contains 22 questions, each describing five levels (global score range from 0 = low burden to 88 = high burden) to measure the burden of care for caregivers.^16,17^ At last, open fields were added for advices to other parents. For the complete surveys, see Supplementary Material part 2.

#### Conduction of the surveys

All young adults with DMD were asked to fill out a short general part on participant characteristics and general statements. Next, they were asked to choose the their top three most important topics from a topic list. On these self-chosen topics they were asked to complete specific questions and statements as described above. After completing the three most important topics, the participants could voluntarily complete additional topics.

#### Data collection and statistical analyses

A digital and validated database (Castor EDC) was used to design the digital survey and for the collection of the completed surveys. Data were anonymized and handled according to guidelines for good clinical practice. This study was approved by the Medical Ethics committee for Research Involving Human Subjects Region Arnhem and Nijmegen, the Netherlands (No 2021-7335).

Descriptive statistics were used to summarize patient characteristics, the importance of each topic for each subgroup (men with DMD and their parents), and scores on the statements. Mean (SD) and median (range) were used for continuous variables depending on their normal distribution. Frequencies (percentages) used for categorical variables. The data were assessed for normal distribution. Differences on statements were explored between the different age categories (16–24 years, ≥ 24 years). Data for males older than 30 years is also described as a subgroup since this is an exception group in participation and care compared to adolescents and young adults. Spearman's correlations were used to analyze associations between the ZBI scores of parents and the age of the participants. Statistical analyses were carried out using SPSS version 25.0 (IBM, Inc., Armonk, New York).

## Results

### Participant characteristics

The surveys were completed by 43 participants with DMD and 30 parents, see Tables 1 and 2. First we will present the results of the adults with DMD, then we will present the results of the survey for the parents. All participants were males and non-ambulatory. The mean age of the adults with DMD was 24.1 years (SD 6.5), although also 16- and 17 year old participants were included, we will call them ‘adults’ in the result section. Majority of the adults with DMD lived with their parents and until 30 years, most of the care was provided by parents. Above 30 years, a median of 20 h of care per week was given by external care givers. Twenty-five percent of the adults (n = 11) with DMD followed special primary education, and 28% (n = 12) followed special secondary education. Nineteen percent of the adults (n = 8) started in regular education and moved to special education. Seven percent of the adults with DMD (n = 3) started in special education and moved to regular education. Sixty-three percent of the adults with DMD (n = 27) followed postsecondary education. Above 24 years of age, 39% (n = 7) has no occupation, 22% (n = 4) does volunteer work, and 33% (n = 6) has a paid job.

**Table 1.** Characteristics of the adolescents and adults with DMD who completed the questionnaire.

	Total group n = 43	Age <24 years n = 25	Age ≥ 24 years n = 18
Age years,			
Mean (SD)	24.1 (6.5)	20 (2.1)	29 (6.2)
Living situation (n/ %)			
- with parents	34 (79)	22 (88)	12 (67%)
- with parents, separate space	2 (5)	1 (4)	1 (6)
- alternating with parents and institution	2 (5)	1 (4)	1 (6)
- institution	3 (7)		3 (17)
- with others	1 (2)	1 (4)	
- other, ..	2 (4)	1 (4)	1 (6)
Environment (n, %)			
- Urban	25 (58)	13 (52)	12 (67)
- Rural area	18 (42)	12 (48)	6 (33)
Education level (n)*			
- regular primary education	23	13	10
- switch to special	8	5	3
- special primary education	11	8	3
- switch to regular	3	3	0
- regular secondary education	31	24	18
- special secondary education	12	8	4
- postsecondary education (vocational/ higher education)	27	13	14
Work (n/ %)			
- no	27 (63)	20 (80)	7 (39)
- Yes, voluntary work	7 (16)	3 (12)	4 (22)
- Yes, employed	8 (19)	2 (8)	6 (33)
- Yes, self-employed	1 (2)		1 (6)
Care by parents per day (Hours, Median, range)	7 (0–24)	10 (1–24)	9 (0–24)
Care by external caregivers per day, (Hours, Median, range)	3 (0–24)	3 (0–24)	11 (0–24)

* multiple answers possible, participants reported the current educational level and the completed education level. In some cases, two levels of secondary education were reported separately.

**Table 2.** Characteristics of the parents who filled in the questionnaire.

	Total group n = 30
Age parent years, mean (SD)	56.0 (6.2)
Age son with DMD years, mean (SD)	22.9 (5.9)
Living situation son n (%)	
- with parents	25 (83)
- partly with parents	1 (3)
- institution/ independent	4 (13)
Family situation parent n (%)	
- together with partner and children	21 (70)
- together with partner	6 (20)
- Single	3 (10)
Environment, n (%)	
Rural	10 (33)
Urban	20 (67)
Educational level parent n (%)	
- primary education	3 (10)
- secondary education	10 (33)
- postsecondary education (vocational/ higher education)	16 (48)
Work parent n (%)	
- no	9 (30)
- Yes, voluntary work	2 (7)
- Yes, employed	15 (50)
- Yes, self-employed	4 (13)
Zarit burden index (ZBI)* Mean (SD)	34.1 (10.5) Range 16–59

* ZBI measures the experienced care burden form 0 (no burden) to 88 (maximal burden).

### Descriptive analyses (young) adults with DMD survey

#### General statements (all participants)

**Figure 1.**
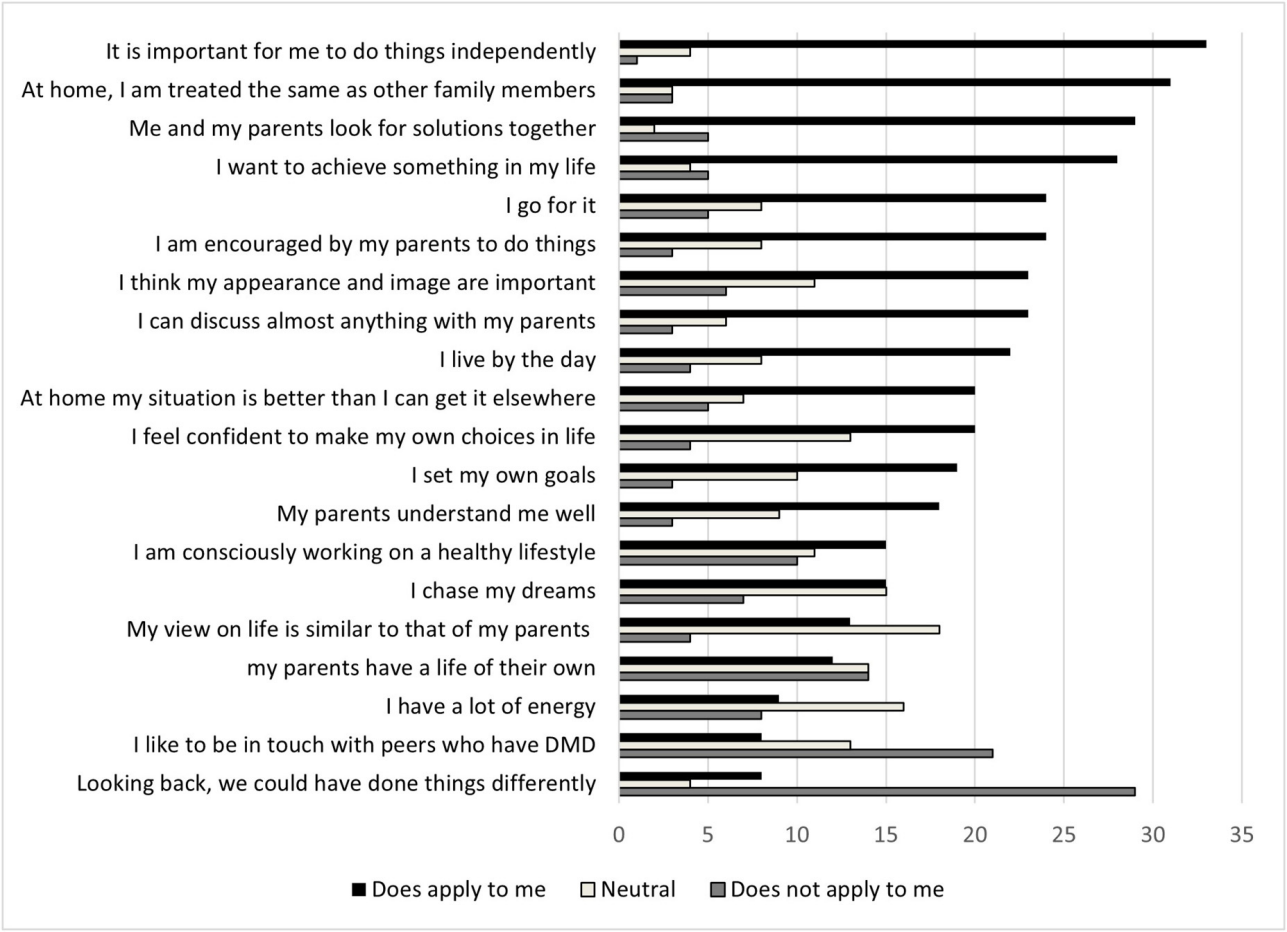
General statements; all participants reported if the statement does or does not apply for them, or if they do not have an opinion on this (neutral). The statements were ranked according to most reported ‘does apply to me’.

During the general part of the survey all participants with DMD scored on the 20 statements, see Figure 1. The statements below were reported most frequent as “Does apply for me”.

‘It is important for me to do things independently’
‘At home, I am treated the same as other family members’
‘Me and my parents look for solutions together’
‘I want to achieve something in my life’

The statement which did not apply for most participants (n > 25) was: ‘Looking back, I would have done things differently’. Analyzing this statement in the different age categories 80% (n = 20) of participants younger than 24 years reported it did not apply to them, and for 44% (n = 8) of participants above 24 years of age this statement did applied to them. For “I can discuss almost anything with my parents”, 84% (n = 21) of the participants under de 24 years reported this statement applied to them, and 11,1 (n = 1) of participants above 24 years of age reported this did not apply. The statement “At home my situation is better than I can get it anywhere else” was supported by the majority, 84% (n = 21) of the younger participants under 24 years of age, which was not supported by 22,2% (n = 4) participants above 24 years of age.

#### Topics with the highest scores

**Figure 2.**
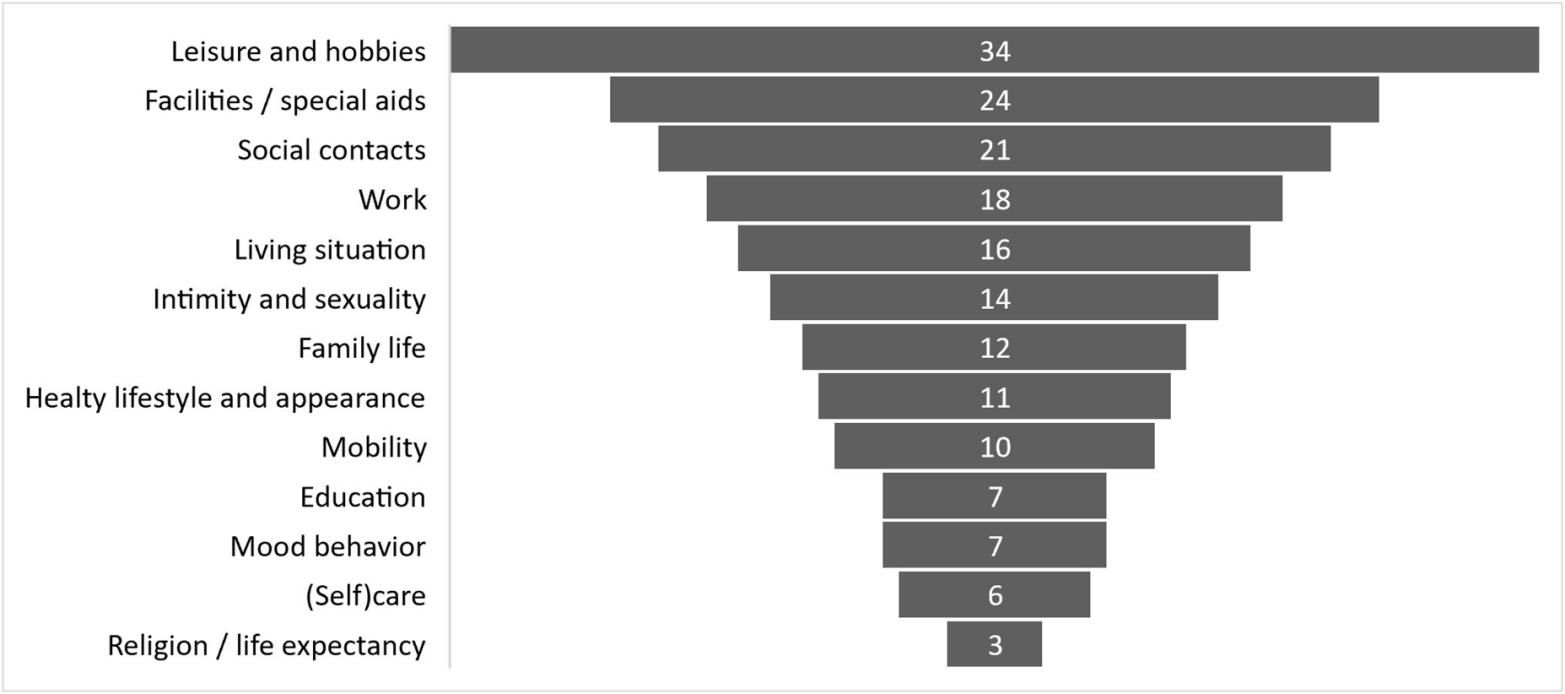
Most important topics that affect life choices reported by the participants with DMD. In total 183 topics were completed by the 43 adults with DMD. The percentages describe the proportion of participants who reported the topic in their top 3.

After completing the general part, the participants were asked to score a top three in topics and complete the questions on this topic. One adult with DMD completed two topics, 46% (n = 20) of the adults with DMD scored three topics, and 51% (n = 22) scored additional topics with a maximum of eight topics by two adults with DMD. The total of 183 topics which were completed by the participants with DMD are displayed in Figure 2. In the next section we will illuminate the five most chosen topics in the total group: ‘leisure and hobbies’, ‘facilities and special aids’, ‘social contacts’, ‘work’, and ‘living situation’. For the group aged above thirty we see that ‘work’ has increased in the priority list to a second place, ‘social contacts’ decreased to a fifth place and ‘relationships and intimacy’ had a third place in the priority list.

For ‘leisure activities’, completed by 34 participants, 85% (n = 29) reported to be satisfied to very satisfied with their current situation. This means that the gap between their current and desired situation is relatively small. Important activities were gaming (91%, n = 31), watching television (73%, n = 25), going out for dinner (68%, n = 23), visiting friends (68%, n = 23) and family (50%, n = 17), going on holiday (53%, n = 18), sports (44%, n = 15) and cultural activities (44%, n = 15). Important factors, that were reported to be of influence on leisure activities were: outdoor mobility, aids and facilities, assistance of parents and friends, energy, and time.

The topic ‘aids and facilities’ was chosen as important topic by 24 participants, of which 50% (n = 12) reported to be satisfied or very satisfied with their current aids and facilities. The desire to have influence on which type of aids and facilities were prescribed, was under scribed by 92% (n = 22), 63% (n = 15) of them experienced the ability to have the desired influence. Important success factors that the participants reported were: good knowledge of facilities (by providers, care professionals, and themselves), financial possibilities, timely intervention, involving an occupational therapist, and adequate communication.

Sixty-two percent of the participants (n = 21) completed ‘social contacts’, 61% (n = 13) was satisfied or very satisfied with their current social contacts. All participants had the desire to have contact with others, 76% (n = 16) experienced the ability to do so. Two patients reported the desire to have contacts with other people with DMD. Important factors, that were reported to be of influence on this topic were the accessibility, outdoor mobility, self-confidence, and leisure activities. Factors which were not of influence according to the participants were parents, work, and religion. Online contacts were seen as a valuable alternative.

Fifty-three percent of the participants (n = 18) completed ‘work’ as one of the most important topics, of which 78% (n = 14) reported that they want to work, and 38% (n = 7) experienced the ability to work. Reported motivation for working was to be able to mean something for others, and having a daily routine. Factors influencing work were the physical abilities, travel distance/ mobility, confidence regarding ability to work . Participants scored neutral on factors of importance for the labor market, fatigue, and the level of education.

Eighty-seven percent (n = 14) of the participants who completed ‘living situation’were satisfied or very satisfied. Sixty-two percent (n = 10)had the desire to live independently, of them 40% (n = 4) were able to live independent. Factors which were reported to be of influence were the availability of suitable homes and the availability of care givers. Factors reported not to have influence on making choices on living situation were parents, financial situation, mobility, or an example of independent living peers.

#### Highlights from other topics

*‘*Intimacy’ was completed by 41% (n = 14) of the participants. Having an intimate relation was considered possible by 71% (n = 10) desired by all of them (100%). Seventy-nine percent (n = 11) reported that they do not want to talk with their parents about this topic.

All participants who scored ‘family life’ as important (28%, n = 12) were also satisfied with the support from their family. Most of them (83%, n = 10) reported that their parents know them best and 58% (n = 7) need their parents for daily activities. Three participants have plans to start their own family.

Having a healthy lifestyle was desired and reported as possible by all participants who completed this topic (26%, n = 11), with exception of sufficient exercise. This was desired by seven participants, while none of them were able to do this.

Of the 23% (n = 10) of the adults who completed ‘mobility’, 70% (n = 7) were very satisfied or satisfied. The support of parents was scored as very important for a sufficient outdoor mobility. Also financial possibilities, and support in transportation schedule is of great influence. Seventy percent (n = 7) reported that wheelchair use is well integrated in our society.

Sixteen percent (n = 7)of the adults with DMD completed ‘education’, 57% (n = 4) of them reported to be satisfied or very satisfied with the education which they followed. Friends, educational level, and indoor- and outdoor mobility are important in making choices on education.

Seventy-one percent (n = 5) of the adults with DMD who completed ‘mood and behavior’ (n = 7) experienced problems with mood or behavior. 86% reported to have had professional help with their problems.

The ‘care’ topic, which was completed by 14% (n = 6) of the adults with DMD showed that care and the organization of care mainly dependents on parents.

No significant differences in reported importance of the topics between the different age categories.

### Parent survey

Table 2 summarizes the characteristics of the 30 participating parents. The living situation of their child with DMD was in the majority of the cases (83%, n = 25) with their parents. The majority of the parents lived together with a partner and their children (70%, n = 21) and was employed (70% n = 21).

#### ZBI

**Figure 3.**
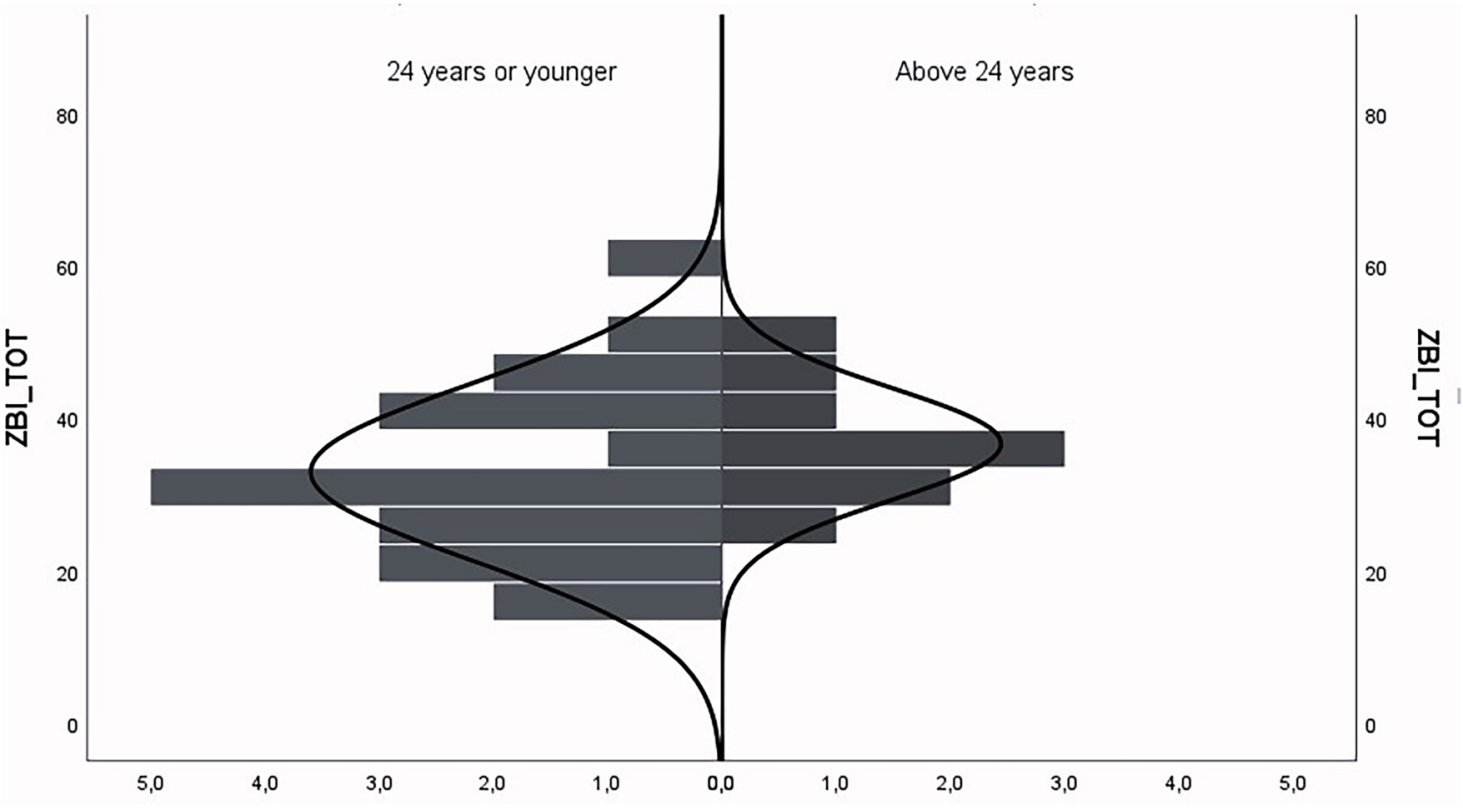
ZBI scores set out in different age categories. The y-axis represents the ZBI measures the experienced care burden form 0 (no burden) to 88 (maximal burden). The x- axis represents the amount of parents.

The ZBI reported by the participating parents ranged between 16 and 59, with a mean ZBI of 34.1. No significant correlation was found between the ZBI and the age of the parents, nor the age of the son with DMD. Figure 3 shows the raw data of the ZBI scores below and above 24 years of age, as 24 is defined as the end of adolescence.^18^ Within the ZBI the highest scores (high burden) were reported in; the time, social contacts, and privacy available for the parents themselves, worries about the future, dependency of their son, and health issues related to care of their son. The lowest scores were reported on being uncomfortable with their son and the wish to outsource care for their sons.

#### General statements

**Figure 4.**
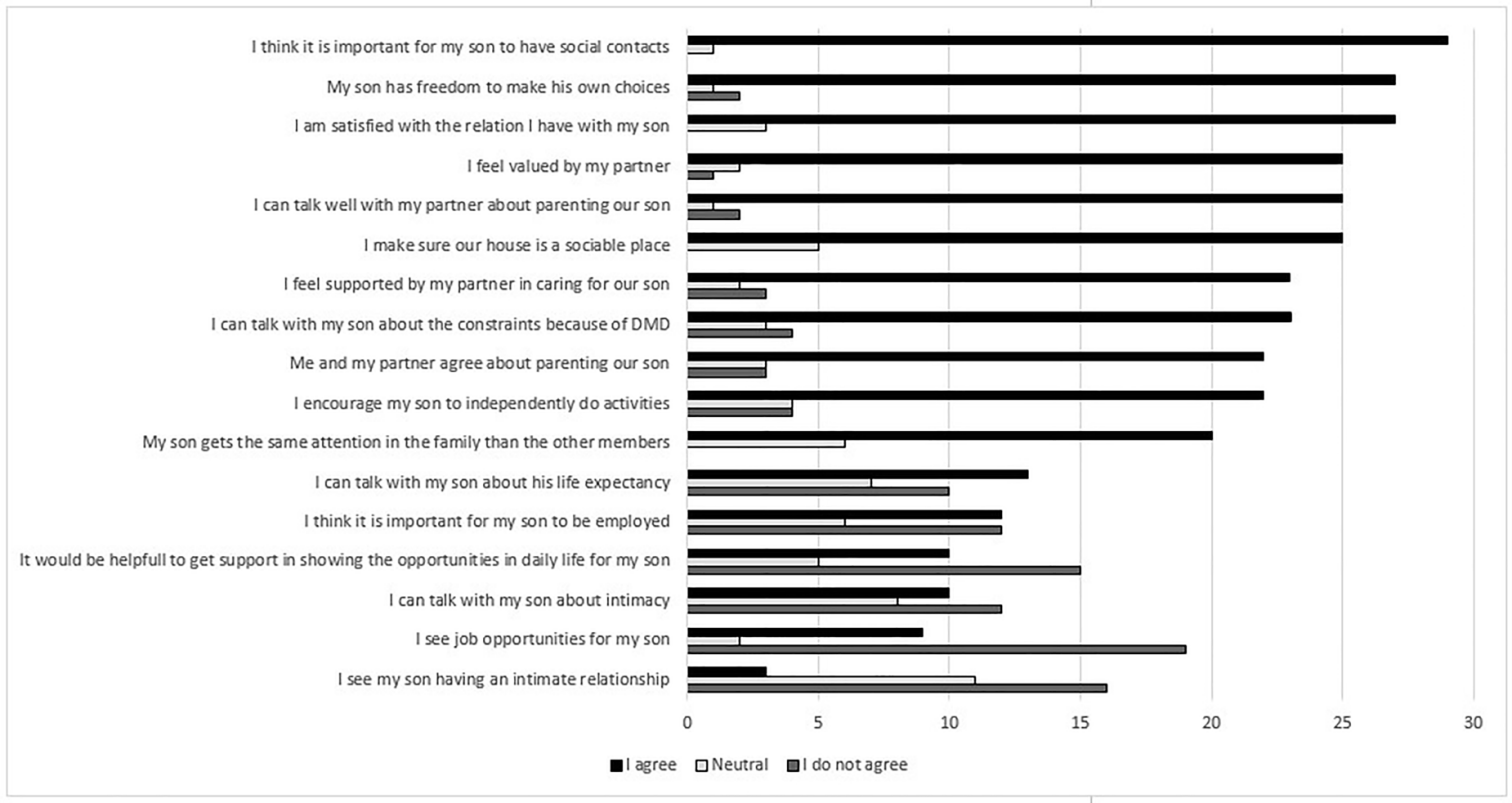
General statements; all parents reported if they did, or did not agree with the statement, or if they were neutral. Ranked according to highest score (i.e., ‘I agree’).

The general statements that were submitted to the parents are summed up in Figure 4. The statements that most parents agreed on (n > 25) were:

‘It is important for my son to have social contacts.’
‘My son has freedom to make his own choices.’
‘I am satisfied with the relation I have with my son.’

Statements that most parents did *not* agree (n > 15) were:

‘I see job opportunities for my son.’
‘I see my son have an intimate relationship.’

#### Parent opinions on the most completed topics by adults with DMD

Parents are less satisfied with the ‘leisure activities’ and ‘social contacts’ of their sons; only 17% (n = 5) of the parents reported to be satisfied or very satisfied, 77% (n = 23) reported they find leisure activities very important. Considering their own leisure activities: 23% (n = 7) of the parents were able to carry out their hobbies as desired. Being away from home for a longer period of time appeared to be a barrier. Parents reported to have contact with other parents of sons with DMD.

Fifty- seven percent (n = 7) had problems in applying for facilities. Twenty-three percent (n = 7) were satisfied or very satisfied with this process. The most important factors in arranging aids and facilities according to parents were the advice of the care professional (90%, n = 27), having control over the application (87%, n = 26), and knowledge and experience of the care professionals (80%, n = 24). Four parents (13%) reported they would want their son to arrange the aids and facilities himself.

Forty percent (n = 12) of the parents reported that they found it important their son is employed, 30% (n = 9) also sees job opportunities for their son. Sixty-three percent (n = 19) of the parents were very dissatisfied regarding the job opportunities. As for their own employment; 63% (n = 19) of the parents wanted to be employed, 95% (n = 18) of them experienced the ability to be employed. The most important factors for being employed themselves were contacts with colleagues (70%, n = 21), having your own life (67%, n = 20), and for financial reasons (60%, n = 18). Of the 17% (n = 5) of the parents of which their son lived (partly) independently, 60% (n = 3) of them were very satisfied with the living situation. Parents reported that finding a suitable place for their son to live is not easy.

#### Comparison between participants with DMD and their parents

Fifteen surveys were completed by parents of the adults with DMD who also completed the survey and could be compared. No additional remarks could be concluded from this comparison.

#### Recommendations for other parents

Several parents emphasized that it is important for parents to maintain activities outside the family setting, alone or as partners. Besides, they advised to start early with the delegation of care tasks to external care givers. Regarding facilities and special aids, clear recommendations were given on having a firm and (pro)active attitude in the process. Moreover it was advised by parents in the survey to collect professional advice and information, preferable by persons who are familiar with the family situation. Concerning work and living situation, the most overlying advice from the parents was to be aware that there is a choice and it is important to regard future perspectives. In the more advanced disease stages, the possibilities to live independently are scarce, several parents were speculating about a collective initiative to combine care and living for adults with DMD.

## Discussion

This study explored the desired participation and choices for participants with DMD during transition and adult life, including barriers and facilitators. Besides, the view and role of parents in this process was explored.

The majority of the participating adults with DMD reported that they want to have an independent life. They scored ‘leisure activities’, ‘social contacts’, and ‘facilities and aids’ as most important topics of influence in reaching an independent life, which overlaps with results from a previous study on participation in DMD adults.^11^ Satisfaction was high for conducting leisure activities, however, satisfaction was lower for social contacts and facilitating aids, in which a gap exists between the current and desired situation. Important factors of influence on these topics were accessibility, outdoor mobility, adequate care facilities, self-confidence, adequate knowledge of professionals and caregivers, and support of parents. The parents who participated in the current study saw limited opportunities about accessing facilities and aids, job opportunities, and their son having an intimate relationship. Concerning their own participation, 60% of the parents were employed, but only 23% of the parents could carry out their leisure activities as they desired.

The majority of the participants of our study lived with their parents (79%), which is a high percentage compared to a Danish study on participation of adults with DMD;^9^ 72.9% of the Danish population above 24 years of age lived alone which largely exceeded the independent living situation of the participants from the current study. All participants in the Danish population had a personal assistant and owned a disability van. We think that appropriate support and facilities can cover a great part of the gap to desired independent participation.

Outdoor mobility, being dependent on parents, and suitable facilities were frequently-mentioned factors which were of influence on the ability to participate and live independently in our study.

Especially the older group of our study population (>30 years) reported that they were not able to live the independent live they desire. Possibly, older participants have more experience and increased desire for an independent life compared to the younger participants, or the younger participants might be more able to live an independent life due to the growing attention for transition in (health)care.

For education and employment, a much larger percentage was employed in the current study compared to the previous studies.^2,9^ It is possible that there is a selection bias and the higher educated people with DMD completed the survey and emphasized the importance of this theme. Our results showed that the possibilities for education and employment exist, but only few adults with DMD were able to utilize these opportunities.

Although adults were (very) satisfied with their leisure activities (in line with previous literature^9,19^), their parents were less content with these daytime activities. As this is the desired situation for the adults with DMD and it suits their physical abilities and energy level, this could be the optimal situation. We have learnt that people with DMD in general are satisfied with their health related quality of life.^20,21^ We wonder if this is because they are indeed happy with their current situation, of if they are not aware of additional possibilities in participation.

As for mood and the need for intimate relationships, this was addressed less often than we would expect from the pilot interview phase of this study. Because we asked the participants to choose three topics which were most determining in living the desired life, it is possible that other topics were more important to the participants. However, a very recent study showed that sexual health should have more attention as it is hard for persons with DMD to talk about this, and lots of barriers exist^22^

The mean score of the caregiver burden in the current study, measured with the ZBI, was slightly higher than in a previous study on caregiver burden in the DMD population (34.1 compared to 29), and comparable with other neuromuscular diseases.^17^ The current study showed that in many aspects adults with DMD were depending on their parents, for example in outdoor mobility or caregiving, in participating in social contacts and employment. The high caregiver burden might be explained by the fact that parents tend to put their own needs aside and most of them cope by a ‘present moment focus’.^19^ Besides, burden is also dependent on the access and expenditure of the necessary accommodations and assistive devices.^20^

The strength of this study was the bottom-up strategy; the adults with DMD and their families were the starting point of this project. Many topics identified in the interviews were also identified in the survey, but there were also differences. Intimacy was addressed more openly as a desire in the interviews compared to the survey. Also the need for role models and the use of external care givers were more often addressed during the interviews, than in the survey. It showed that a dialogue revealed more dept on topics than a survey.

The translation from the interviews to the survey was a challenge, because a broad pallet of rich information had to be translated in an unambiguous survey. The statements were chosen from the interviews by the expert group. The statements represent goals in live and the relations with the parents. Although, these choices were made subjectively and other issues could be equally important, we think that the representatives in the expert group, including health care professionals, qualitative researchers, external care givers, and experts by experience, were able to extract the key point issues representative for the (young) adult DMD community. The findings of this study have to be seen in light of the limited size of the population. We were hoping to receive a high response rate. However, because of the already extensive burden on people with DMD and their parents, participating in research is challenging.^11,23^ This may have led to a selection bias, in which participants who felt having time and those with higher cognitive abilities have completed the survey more likely than others. To minimize the burden, the participants were asked to complete only three topics instead of the whole survey. This gave a good insight in the importance of the different topics and the open fields were completed extensively. The disadvantage of this approach was that the power for statistical sub-analyses was lacking, however, descriptive analysis enable us to identify factors of influence on living the desired life with DMD. Besides, because participants could complete the survey anonymously, genetic test for DMD was not confirmed.

Our findings have provided new insights in the facilitators and barriers in living the desired life for young adults with DMD. Environmental factors and social services appeared to be of great influence on the opportunities to participate for people with DMD. The life with DMD is fortunately lived outside the hospital and rehabilitation centers. Health care professionals have to collaborate with partners from the patient advocacies, public authority, and business community to provide medical knowledge on disease specific needs and to create awareness on the possibilities. As previously described; more attention is needed on opportunities and experiences instead of focusing only on the person's limitation and need for skill development.^24^ Besides, unambiguous information about social services, facilities, and aids can help young adults with DMD as well as their parents to be more empowered in making choices in live. Finally, the dilemma of parents, existing of the lack of time for their own activities and not wanting to outsource care for their son, needs more attention and support.

The insights and recommendations from this study are important to raise awareness among clinicians and are intended to guide (young) adults with DMD to enlarge their control over the transition process and empower them in utilizing the life they desire. Moreover, appropriate support and facilities can cover a great part of the gap to desired independent participation. At last, it is important that parents are guided to be more aware of the different roles and choices they have in this process.

## Supplemental Material

sj-docx-1-jnd-10.1177_22143602251324847 - Supplemental material for Factors affecting desired participation in transition to an adult life with Duchenne muscular dystrophy (DMD) Supplemental material, sj-docx-1-jnd-10.1177_22143602251324847 for Factors affecting desired participation in transition to an adult life with Duchenne muscular dystrophy (DMD) by Laura JB Merkenhof, Yvonne Veenhuizen, Elizabeth Vroom, Greet Sterenberg, Wendy CHM Hesseling, Jan T Groothuis, Edith H Cup and Saskia LS Houwen- van Opstal in Journal of Neuromuscular Diseases

sj-docx-2-jnd-10.1177_22143602251324847 - Supplemental material for Factors affecting desired participation in transition to an adult life with Duchenne muscular dystrophy (DMD) Supplemental material, sj-docx-2-jnd-10.1177_22143602251324847 for Factors affecting desired participation in transition to an adult life with Duchenne muscular dystrophy (DMD) by Laura JB Merkenhof, Yvonne Veenhuizen, Elizabeth Vroom, Greet Sterenberg, Wendy CHM Hesseling, Jan T Groothuis, Edith H Cup and Saskia LS Houwen- van Opstal in Journal of Neuromuscular Diseases

## References

[1] Mulder RL van der Pal HJH Levitt GA , et al. Transition guidelines: an important step in the future care for childhood cancer survivors. A comprehensive definition as groundwork. Eur J Cancer 2016; 54: 64–68. 26735352 10.1016/j.ejca.2015.10.007

[2] Peay HL Do BT Khosla N , et al. Role attainment in emerging adulthood: subjective evaluation by male adolescents and adults with Duchenne and Becker muscular dystrophy. J Neuromuscul Dis 2022; 9: 447–456. 35275556 10.3233/JND-210709 PMC9126318

[3] Passamano L Taglia A Palladino A , et al. Improvement of survival in Duchenne muscular dystrophy: retrospective analysis of 835 patients. Acta Myol 2012; 31: 121–125. 23097603 PMC3476854

[4] Broomfield J Hill M Guglieri M , et al. Life expectancy in Duchenne muscular dystrophy: reproduced individual patient data meta-analysis. Neurology 2021; 97: e2304–e2e14. 10.1212/WNL.0000000000012910 PMC8665435 34645707

[5] Landfeldt E Thompson R Sejersen T , et al. Life expectancy at birth in Duchenne muscular dystrophy: a systematic review and meta-analysis. Eur J Epidemiol 2020; 35: 643–653. 32107739 10.1007/s10654-020-00613-8 PMC7387367

[6] Birnkrant DJ Bushby K Bann CM , et al. Diagnosis and management of Duchenne muscular dystrophy, part 3: primary care, emergency management, psychosocial care, and transitions of care across the lifespan. Lancet Neurol 2018; 17: 445–455. 29398641 10.1016/S1474-4422(18)30026-7 PMC5902408

[7] Yamaguchi M Sonoda E Suzuki M . The experience of parents of adult sons with Duchenne muscular dystrophy regarding their prolonged roles as primary caregivers: a serial qualitative study. Disabil Rehabil 2019; 41: 746–752. 29172756 10.1080/09638288.2017.1408148

[8] Donaldson A Guntrum D Ciafaloni E , et al. Achieving life milestones in Duchenne/Becker muscular dystrophy: a retrospective analysis. Neurol Clin Pract 2021; 11: 311–317. 34484931 10.1212/CPJ.0000000000000970 PMC8382433

[9] Rahbek J Werge B Madsen A , et al. Adult life with Duchenne muscular dystrophy: observations among an emerging and unforeseen patient population. Pediatr Rehabil 2005; 8: 17–28. 15799132 10.1080/13638490400010191

[10] Handberg C Werlauff U Højberg AL . Perspectives on everyday life challenges of Danish young people with Duchenne muscular dystrophy (DMD) on corticosteroids. Glob Qual Nurs Res 2022; 9: 23333936221094858. 35493771 10.1177/23333936221094858 PMC9052227

[11] Houwen-van Opstal SLS Heutinck L Jansen M , et al. Occurrence of symptoms in different stages of Duchenne muscular dystrophy and their impact on social participation. Muscle Nerve 2021; 64: 701–709. 34453345 10.1002/mus.27406 PMC9292483

[12] Lindsay S Cagliostro E McAdam L . Meaningful occupations of young adults with muscular dystrophy and other neuromuscular disorders. Can J Occup Ther 2019; 86: 277–288. 31096763 10.1177/0008417419832466

[13] Lindsay S McAdam L Mahendiran T . Enablers and barriers of men with Duchenne muscular dystrophy transitioning from an adult clinic within a pediatric hospital. Disabil Health J 2017; 10: 73–79. 27769758 10.1016/j.dhjo.2016.10.002

[14] Hamdani Y Mistry B Gibson BE . Transitioning to adulthood with a progressive condition: best practice assumptions and individual experiences of young men with Duchenne muscular dystrophy. Disabil Rehabil 2015; 37: 1144–1151. 25190331 10.3109/09638288.2014.956187

[15] Gibson BE Mistry B Smith B , et al. Becoming men: gender, disability, and transitioning to adulthood. Health (London) 2014; 18: 95–114. 23456143 10.1177/1363459313476967

[16] Schreiner AS Morimoto T Arai Y , et al. Assessing family caregiver's mental health using a statistically derived cut-off score for the Zarit Burden interview. Aging Ment Health 2006; 10: 107–111. 16517485 10.1080/13607860500312142

[17] Landfeldt E Lindgren P Bell CF , et al. Quantifying the burden of caregiving in Duchenne muscular dystrophy. J Neurol 2016; 263: 906–915. 26964543 10.1007/s00415-016-8080-9 PMC4859858

[18] Sawyer SM Azzopardi PS Wickremarathne D , et al. The age of adolescence. Lancet Child Adolesc Health 2018; 2: 223–228. 30169257 10.1016/S2352-4642(18)30022-1

[19] Rahbek J Steffensen BF Bushby K , et al. 206th ENMC international workshop: care for a novel group of patients - adults with Duchenne muscular dystrophy Naarden, The Netherlands, 23–25 May 2014. Neuromuscul Disord 2015; 25: 727–738. 26099652 10.1016/j.nmd.2015.05.005

[20] Pangalila R . Quality of life in Duchenne muscular dystrophy: the disability paradox. Dev Med Child Neurol 2016; 58: 435–436. 26534893 10.1111/dmcn.12959

[21] Uttley L Carlton J Woods HB , et al. A review of quality of life themes in Duchenne muscular dystrophy for patients and carers. Health Qual Life Outcomes 2018; 16: 237. 30567556 10.1186/s12955-018-1062-0 PMC6299926

[22] Hoskin J Cheetham TD Mitchell RT , et al. Sexual health and fertility in Duchenne muscular dystrophy-an exploratory study. Muscle Nerve 2024; 70: 402–408. 38989790 10.1002/mus.28201

[23] Naarding KJ Doorenweerd N Koeks Z , et al. Decision-Making and selection bias in four observational studies on Duchenne and Becker muscular dystrophy. J Neuromuscul Dis 2020; 7: 433–442. 32925089 10.3233/JND-200541 PMC7902964

[24] Nguyen T Stewart D Gorter JW . Looking back to move forward: reflections and lessons learned about transitions to adulthood for youth with disabilities. Child Care Health Dev 2018; 44: 83–88. 29082531 10.1111/cch.12534

