# Supplementary material for: Factors affecting desired participation in transition to an adult life with Duchenne muscular dystrophy (DMD)
Source: J Neuromuscul Dis. 2025 Mar 3;12(3):353–63. doi: 10.1177/22143602251324847 (PMC13142876; doi:10.1177/22143602251324847)
Supplement: sj-docx-1-jnd-10.1177_22143602251324847 - Supplemental material for Factors affecting desired participation in transition to an adult life with Duchenne muscular dystrophy (DMD) [file sj-docx-1-jnd-10.1177_22143602251324847.docx]

**NEW PERSPECTIVE ON ADULT LIFE WITH DUCHENNE MUSCULAR DYSTROPHY**

***The voice of the boys***

Our perspective were the eyes and voice of (young) adults with DMD, a bottom up approach instead of the regular perspective of care professionals, practitioners or health sciences.

We conducted free single interviews lasting 3 to 4 hours at their home, with their daily routine. If they had plans for the day, we went with them, to the physio, wheelchair hockey, music lesson, whatever came across. We interviewed their parent(s) as well – in most cases seperately – to be able to talk as open and freely as possible. Striking was that all persons we talked to – boys/men and their parents – felt good about telling their story without constraints. It also evoked new insights for themselves and topics they felt needed to be talked about

**What we learned:**

***Everyone is unique.*** Every DMD patient is different (specific genetic disorders, differences in mental status). Progression of the disease is different for each patient. If you have DMD there’s nothing to hold on to, no reference.

A continuous balancing between ***wanting to and being able to***, for DMD patients and their parents. There are different strategies to cope with adult life with DMD.

We found ***Warriors*** (fighting, active, wanting to be part of normal life, searching opportunities). We found ***Realists*** (anticipating, overtaken by reality, focus on coping with life as it is). We found ***Floaters*** (laissez faire, passive attitude, daily routine is leading). We found ***Escapists*** (withdrawing, small world, don’t have to do anything-attitude).

And how do the life strategies of adolescent/adult men fit with the life strategies of their parents? Stimulating taking control or taking over control. There are different scenario’s regarding adult life with Duchenne

***DMD rules the family***; family life is driven by Duchenne. There’s no escape. As time goes by, the dependency of boys/men with Duchenne becomes greater and the burden on parents/caregivers (also siblings) becomes heavier. Duchenne patients are aware that they disrupt family dynamics. Parents become caregivers. The natural flow of an adolescent to rebel against parents is not feasible for a Duchenne patient. It feels oppressive.

In Duchenne families seems to be lots of unspoken dilemmas; asking too much of yourself but not being able to admit it, denying yourself things to prevent extra burden on others, relationships that can't handle the pressure, parents (especially mothers) who take on a full-time care role, physical complaints from parents, burnout.

For most boys and men with DMD the aim is to participate '***as normal as possible'*** in a valid world. Do what your peers do. Adolescent Duchenners (16-20 years old) are confident that they can live as normal as possible and do not (yet) consider that this might not be feasible. As age increases, more and more help and resources are needed to do what you want. It takes lots of efforts to keep (or extend) a social network. It is complicated to connect with the rhythm of life of peers, because nothing can happen spontaneously. Everything must be arranged. For most boys and men, the world is getting increasingly smaller. Social isolation is lurking. A symbiotic relationship might develop between parent and child, which constraints development of one's own identity.

To be able to take control of your life, care must be well organized**. *The task called care*** is a major issue. Arranging all care and facilities requires a lot (usually from the parents). Virtually none of the men with Duchenne we spoke were able to arrange care themselves. Arranging care is perceived to be a nightmare. The care on offer is opaque, there is lots of regulation, you are snowed under by bureaucracy, people experience little understanding, everything takes a long time, it is very frustrating.

Importance of care increases with age, more (specialized) help is needed. Demands on helpers are becoming higher and therefore increasingly difficult to arrange. This creates a great sense of dependence; limitation of personal choice due to fixed times of care and a lot of stress when helpers fall out. For parents (especially mothers) it seems difficult to outsource care. In addition, the PGB has often become a substantial part of the family income. Boys/men with Duchenne who are primarily cared for by their parents find it difficult to accept external care.

The families who did opt for external care indicate that it has improved everyone's life. Parents can be parents again. Sons are mentally relieved because they don't have to constantly rely on their parents.

Care by peers contributes a lot to being able to take control of one's own life; brings social contacts, stimulates and inspires, brings excitement and dynamism.

Healthcare is mainly about physical care. Mental health care seems to be secondary. *'If you have to do that, the end is lost, we already have so much to do.'*

Don't show your weaknesses and don't admit to them. ‘Be a strong mother to your son. Be a strong son to your mother.’

There seems to be a huge drive to be optimistic and positive - in both Duchenne patients and their parents (although several respondents do have mood swings).

If you have DMD, living independently (part of living an adult life) is very difficult. Possibilities are limited, it takes drive, money, and a strong motivation to realize independent living. Most respondents were living at their parents’ home.

Living a ***meaningful life*** is of crucial importance to the boys and men we spoke to. Working, making money, falling in love, having sex, family life. Finding work, an internship or even volunteering is extremely difficult – no matter how many degrees you have. And where and how do you find a date? Gradually, there are more experiences of being rejected in various areas. Undermining self-confidence and ambitions.

Boys/men with DMD are masters of living in the here and now. They don't like to think about the future. A day-to-day strategy is a way to keep a grip on the situation; short-term prospects, looking forward to things happening in short notice. Most of them have a pragmatic and solution-oriented attitude; there is no point in giving up. So how to proceed from here? '*My thumb doesn't work anymore, how to cope?'*

We often heard in the interviews (from Duchnenne boys/men and parents) that, in hindsight, they might have done things different (choice of school, social contacts, etc.).

***Activities*** are mainly concentrated at home; computing, making music, podcasts, drawing, photographing. Wheelchair hockey is (or has been) important to many boys.

Managing energy becomes increasingly complicated when getting older. The online world offers escape and fulfilling pastime. Online you’re normal, a world of your own, social contacts, no interference of parents. Parents are kind of worried about their son spending that much time online.

***Need for intimacy*** is an important aspect in adult life with Duchenne, but difficult to measure in quantitative research. The topic needs the context of a face-to-face conversation. Most of the boys/men we spoke dream of dating, sex, a relationship, a family. Sexuality is perceived to be a subject that needs more attention.

The majority of boys/men with DMD ***don’t like to talk about Duchenne***. The awareness of DMD is low. Outsiders don’t know what it is and cannot assess the seriousness of the situation. Duchenne patients say they are not actively looking for contact with other men with DMD (which is confrontational), but at the same time they also seem to enjoy exchanging experiences if you happen to meet someone with DMD. There is a need for role models or examples of what living an adult life with DMD might look like.

**Conclusions**

1. There is still a lot to do to put adult life with Duchenne on the map

- among DMD patients, their parents, healthcare professionals, employers, society

2. As physical self-reliance decreases, the importance of taking control of life increases

- finding a balance between 'wanting and being able'

- searching for solutions and possibilities

- you don't have to be able to do everything yourself if you are able to manage others

3. Taking control requires courage and endurance

- that needs to be learned and stimulated

- important to be aware of this at an early stage

4. Parents have a crucial role

- recognizing the importance of encouraging their son to take control

- prevent breaking down themselves

5. Facilitating control of life through care solutions and life solutions
