# Supplementary material for: Factors affecting desired participation in transition to an adult life with Duchenne muscular dystrophy (DMD)
Source: J Neuromuscul Dis. 2025 Mar 3;12(3):353–63. doi: 10.1177/22143602251324847 (PMC13142876; doi:10.1177/22143602251324847)
Supplement: sj-docx-2-jnd-10.1177_22143602251324847 - Supplemental material for Factors affecting desired participation in transition to an adult life with Duchenne muscular dystrophy (DMD) [file sj-docx-2-jnd-10.1177_22143602251324847.docx]

**Supplementary material 2: Surveys for (A) adults with DMD and (B) for parents of adults with DMD**

**A. Survey for adults with DMD**

**PART 1 General questions**

How old are you?

What is your living situation:

- - I live at my parents' house - click through purchase/rent
  - I live with my parents in an independent space - click through purchase/rent
  - I live with my parents, but I regularly go to overnight accommodations
  - Own home—click through purchase/rent
  - I live in my own home with others
  - Focus house
  - Dorm room
  - Assisted living group
  - Otherwise..

Living environment:

- City/region with many facilities nearby

- Outside area

Will your living situation change soon? Yes No

If so, what will change? Open question

What type of primary education did you receive? *According to the Dutch scholar system*

- Regular primary education (RO)
- Special primary education (SBO)
- Special education (SO)
- Special secondary education (SVO)
- Started in regular education, switched to special education
- Started in special education, switched to regular education

What level of secondary education did you have? *According to the Dutch scholar system*

- Practical education
- LWOO (learning support education)
- VMBO theoretical learning path (VMBO-T)
- VMBO mixed learning path (VMBO-G)
- VMBO framework vocational learning path (VMBO-K)
- VMBO basic vocational learning path (VMBO-B)
- HAVO
- VWO
- Otherwise ..
  - - Have you completed this education?

Did you follow any training after secondary education? Yes No

If so, what training have you completed? *According to the Dutch scholar system*

- MBO level 1
- MBO level 2
- MBO level 3
- MBO level 4
- HBO bachelor's degree
- HBO Master
- WO; University bachelor's degree
- WO; University master's degree
- Have you completed this education?

Do you work?

- No
- Yes, paid as an employee
- Yes, paid as a self-employed person
- Yes, unpaid

Self-care:

I get…. hours of help from my parents per day

I receive ... hours of assistance from external care providers per day (PBG/ADL assistance/home care)

How can we best inform you about the progress of this project?

-Facebook

-Instagram

- Tiktok

- Snapchat

- Linked In

- E-mail

- Otherwise, namely

Autonomy:

Below are a number of statements. To get to know you better, we would like to ask you to give a rating between 0 and 5.

0 = Not applicable to me at all

5 = Completely applies to me

* My parents encourage me to do things ` 0-5

* I want to achieve something in my life 0-5

* I have a lot of energy 0-5

* My parents have their own lives 0-5

* I chase my dreams 0-5

* I'm going for day by day 0-5

* My parents understand me well 0-5

* I set my own goals 0-5

* My parents work with me to find solutions 0-5

* I can discuss almost everything with my parents 0-5

* In retrospect, we could have done things differently 0-5

* I am treated the same as the rest at our home 0-5

* I am consciously working on a healthy lifestyle 0-5

* I think my appearance and appearance are important 0-5

* I feel confident to make my own choices in my life 0-5

* My view on life is similar to that of my parents 0-5

* I am better off at home than I can get 'outside the house' 0-5

* I would like to have contact with others with Duchenne muscular dystrophy 0-5

* I think it is important that I can continue to do some things independently 0-5

**PART 2: Theme specific questions**

*How important do you find these topics when it comes to making choices in your life:*

1. Facilities/resources 0-10
2. Transport/mobility 0-10
3. Leisure and hobbies 0-10
4. Work (paid/unpaid) 0-10
5. Education 0-10
6. Intimacy and sexuality 0-10
7. (Self)care 0-10
8. Living situation 0-10
9. Mood/ behavior 0-10
10. Religion/spirituality 0-10
11. Family life 0-10
12. Social contacts 0-10
13. Life expectancy 0-10
14. Healthy lifestyle 0-10
15. Appearance/appearance 0-10
16. Other

*Make a top 3 of the points below that have had a major influence on making choices in your life. More questions about these three topics follow.*

1. Aids and facilities
2. Transport/mobility
3. Leisure time and hobbies
4. Work (paid/unpaid)
5. Education
6. Intimacy and sexuality
7. (Self) care
8. Living situation
9. Mood/ behavior
10. Religion/spirituality & Life expectancy
11. Family life
12. Social contacts
13. Healthy lifestyle & Appearance

**Aids and facilities**

How did you obtain facilities and resources? Multiple answers possible:

- Via the municipality/WMO

- Through health insurance

- Bought with my own money

- Bought myself with budget PGB

- Bought myself with a subsidy/ crowdfunding

How satisfied are you with the provision of facilities? 0 not satisfied 5 very satisfied.

(space for explanation)

Would you like to have more influence when applying for a facility? Yes No

Do you ever experience problems when applying for facilities? Yes/no

Statements:

From interviews with boys and men with Duchenne, we have learned that there can be a difference between what you want and what you can do. Below are a few statements about what you would like and what you can do in practice with regard to arranging facilities.

0 = Not applicable to me at all

5 = Completely applies to me

I would like to determine which provisions I will take

I can decide for myself which provisions I take

I want to apply for a facility myself

I can apply for a facility myself

General statements (score 0-5)

I can find information to properly weigh the pros and cons of the facilities for myself

My facilities are easy to use

The people I work with for a facility involve me in choices for the facilities

The people who help me with the application/purchase of facilities understand what I need

What do you need to make a good choice for a suitable facility? Multiple answers possible:

Which factors often influenced receiving facilities?

- 1. Knowledge about options, for example via an occupational therapist, the support grant, or websites)
  2. Knowledge about the advantages and disadvantages of the facilities
  3. Financing options
  4. Knowledge about who I can request which facilities from
  5. Supplier delivery times
  6. Knowledge of therapists/care providers involved to advise on finding a suitable facility
  7. Examples and tips from other men/boys with Duchenne
  8. Knowledge from the supplier about suitable facilities
  9. Accessibility of those involved in the application for facilities
  10. Communication between the various parties involved in the application for facilities
  11. Otherwise, namely…

What tips do you have for someone else with Duchenne when applying for services? ……………

**Transport​/mobility**

What means of transport do you use? Multiple options possible

- Manual wheelchair
- Manual wheelchair with electric drive
- Handbike
- Mobility scooter
- Electric wheelchair
- Own bus, drive yourself
- Own bus, someone else drives

- Parents

-Friends​

- someone else (including healthcare providers, PGB)

- Cab
- public transport
- Otherwise, namely…

How satisfied are you with your means of transport? 0 not satisfied 5 very satisfied.

(space for explanation)

Would you like more input on transportation options? Yes No

Do you have problems arranging means of transport? Yes No

From interviews with boys and men with Duchenne, we have learned that there can be a difference between what you want and what you can do. Below are a few statements about what you would like and what you can do in practice with regard to transport.

(score 0-5)

I want to decide when I go out

I can decide when I go out

I want to decide for myself where I go

I can decide where I go

I want to decide for myself which means of transport I use

I can decide for myself which means of transport I use

General statements (score 0-5)

Wheelchair use is well integrated into our society

Which points are influential when arranging transport?

- Knowledge about facilities/adapted driving
- Knowledge about taxi transport
- Knowledge about public transport options
- Knowledge about obtaining a disabled parking card
- Financing options
- Parental assistance for driving/parental involvement?
- Help from friends for driving
- Deployment of external emergency services for driving
- Support in planning transport/making appointments with taxi/public transport
- Otherwise, namely…

What tips do you have for someone else with Duchenne regarding transportation? ……………

**Leisure/hobbies**

What are important leisure activities or hobbies for you?

- Gaming/laptop/computer
- Watching movies/TV
- Meeting up with friends
- Meeting with family
- To play sports
- Read
- To travel
- Holidays
- Making/listening to music
- Going to a restaurant/cafe/terrace
- Going out
- Cultural pursuits
- Being creative (drawing, designing, photographing)
- Otherwise, namely…

From interviews with boys and men with Duchenne, we have learned that there can be a difference between what you want and what you can do. Below are a few statements about what you would like and what you can do in practice with regard to your free time.

I want to decide for myself what I will do in my free time

I can decide for myself what I will do in my free time

I want to go on vacation

I can go on holiday

Other statements:

I can do all the activities that interest me

What do you find important in your free time activities and hobbies:

- Being fanatic is 0-10
- Achieving ambition 0-10
- Contact with others 0-10
- Daytime activities 0-10
- Fitness 0-10
- Distraction 0-10
- Sense of equality 0-10
- Immersion/absorption in another world 0-10
- Fun 0-10
- Relaxation 0-10
- Meaning 0-10
- Just participate like anyone else 0-10
- Otherwise, namely…

How important is pursuing hobbies to you? 0 = not important 10 = very important

How satisfied are you with doing your hobbies? 0 = not satisfied 5 = very satisfied

(space for explanation)

Which points influence the performance of your hobbies? Multiple choices possible

- Transport
- Services
- Time
- Energy to perform activities
- Help parents
- Help from friends
- Help from therapists
- Contact with fellow sufferers
- Financing options
- Presence of foundations that arrange trips
- Otherwise, namely …

Do you have any tips for someone else regarding pursuing leisure activities and hobbies? …..

**Work​**

*In this topic we speak of work, which means different things. This includes paid work, unpaid work, volunteer work and/or daytime activities.*

How important is work to you? 0 not important – 10 very important

What did you want to become as a child?

From interviews with boys and men with Duchenne, we have learned that there can be a difference between what you want and what you can do. Below are a few statements about what you would like and what you can do in practice with regard to arranging work

I want to work

I can work

I want to earn money by working

I can make money working

General statements (score 0-5)

Working gives me a rhythm

Working fills my day

I want to mean something to someone else through the work I do

I can do work that I like to do

I can do work at the level for which I was trained

Which points influence your choice of whether or not to work? (0 no influence, 10 a lot of influence)

- Labor market/demand for work 0-10
- Physical capabilities 0-10
- Contact with colleagues 0-10
- Fatigue 0-10
- Time investment in care tasks/arranging care 0-10
- Secondary education 0-10
- Travel distance/transport 0-10
- Motivation/work ambitions 0-10
- Friends 0-10
- Family 0-10
- Feeling of certainty that I can handle it 0-10
- Need for meaning 0-10
- Otherwise: …….

Have you had help in making a choice for the type of work or daytime activities? Yes No

What help did you receive in choosing work? Multiple answers possible:

- - School/internship advice
  - Information from rehabilitation team/hospital
  - Advice parents
  - Advice peers
  - Advice fellow sufferers
  - Information meetings
  - Other, namely…

Did you miss help? Yes No

If so, what did you miss afterwards? Multiple answers possible:

- Advice parents
- School/internship advice
- Information from rehabilitation team/hospital
- Advice peers
- Advice fellow sufferers
- Information meetings
- Other, namely…

Do you have any tips for someone else regarding performing work? …..

**Education**

How did you decide on your primary education? Multiple answers possible:

- Advice parents
- Advice Teacher/Individual/school
- Self-chosen
- Rehabilitation center advice
- Advice friends
- Otherwise..

Looking back, are you satisfied with the primary education you received? 0 not satisfied -5 satisfied

(space for explanation)

(only if they answered the general questions that they had completed secondary education)

How did you choose your secondary education? Multiple answers possible:

- Advice parents
- Advice Teacher/Individual/school
- Self-chosen
- Rehabilitation center advice
- Advice friends
- Otherwise..

Are you satisfied with the secondary education you followed? 0 not satisfied -5 satisfied

(space for explanation)

(only if they answered the general questions that they had completed secondary education)

How did you choose your further education? Multiple answers possible:

- Advice parents
- Advice Teacher/Individual/school
- Self-chosen
- Rehabilitation center advice
- Advice friends
- Otherwise..

Are you satisfied with the further education you followed? 0 not satisfied -5 satisfied

(space for explanation)

From interviews with boys and men with Duchenne, we have learned that there can be a difference between what you want and what you can do. Below are a few statements about what you would like and what you can do in practice with regard to arranging education

I want/wanted to follow a course

I can/could follow a training course

I want/wanted to go to a school/course nearby

I can/could go to a school/course nearby

General statements (score 0-5)

I was sufficiently challenged during my school years

I made friends during my school days

I felt *no* different from the others in the class

I still keep in touch with friends from my school days

How important do you think the following points are when it comes to education?

(0 not important, 10 very important)

- Friends/contact with peers 0-10
- Offer of transport to school 0-10
- School nearby 0-10
- Offering the teaching material 0-10
- Individual guidance 0-10
- Offer of therapy 0-10
- Atmosphere 0-10
- Accessibility (wheelchair, lift option, large school) 0-10
- Options for care 0-10
- Otherwise, namely... 0-10

Have you had help in making a choice for education? Yes No

If so, what help have you had?

- Help from teacher/internal/outpatient supervisor
- Assistance from a remedial educationalist/psychologist
- Help otherwise, namely: …
- Advice from parents
- Information about different types of education
- Tour of various schools
- Participation days at different schools
- Experience of other peers
- Other boys' experience with Duchenne
- Otherwise: …

Which points influenced your choice of school?

Multiple answers possible:

- - Information rehabilitation center/hospital
  - Friends
  - Motivation
  - Appropriate transition from secondary education to further education
  - Care offered at school/possibility of assistance during training
  - Transport to the training
  - Student days
  - Options for independent living/rooming
  - More job opportunities
- Help from teacher/internal/outpatient supervisor
- Assistance from a remedial educationalist/psychologist
- Advice from parents
- Information about different types of education
- Tour of various schools
- Participation days at different schools
- Experience of other peers
- Experience of other boys with Duchenne
- Otherwise, namely..

What tips do you have for someone else with Duchenne regarding education? ……………

**Intimacy and sexuality**

Are you in a relationship and/or are you intimate with someone? Yes No

If so, how did this contact arise?

- Met someone
- Through friends
- Through family
- Through an escort company
- Through internet dating
- Otherwise, namely

If not, do you need intimate contact? Yes No

From interviews with boys and men with Duchenne, we have learned that there can be a difference between what you want and what you can do. Below are a few statements about what you would like and what you can do in practice with regard to intimacy and sexuality.

I want to be in a relationship

I can be in a relationship

I want to have sex

I can have sex

I want to talk to my parents about sex

I can talk to my parents about sex

I want to talk to my friends about sex

I can talk to my friends about sex

I want to have sex at home

I can have sex at home

General statements (score 0-5)

I feel confident about entering into a relationship

I feel confident about having sex

I see myself as a (possible) partner

Which points are important to you with regard to intimate contacts (o not important 10 very important):

- Permanent relationship 0-10
- Living together 0-10
- Sex 0-10
- In-depth conversations 0-10
- Desire/need 0-10
- Experience 0-10
- Paternity 0-10
- Otherwise… 0-10

Do you need help with intimate contacts? Yes No

If so, what help would you like? …

Which points influence you when entering into a relationship and/or intimate contact?

- - Living situation
  - Self confidence
  - Discussion with parents
  - Discussion with friends
  - Religion/spirituality
  - Information about dating
  - Information about escort companies
  - Accessibility areas
  - Physical capabilities
  - Transport
  - Digital possibilities
  - Situations where you encounter others
  - Otherwise..

Do you have any tips for someone else regarding a relationship or intimate contacts? …..

**Care and self-care**

Who do you receive care from? Multiple answers possible

- 1. Parents (incl. foster parents/stepparents)
  2. Brothers Sisters
  3. Partner
  4. Healthcare providers/home care
  5. Care of institution ( Fokus )
  6. Volunteers
  7. Assistance dog
  8. Otherwise..

How many hours of care do you have per day?

1. 0-2 hours
2. 2-4 hours
3. 4-6 hours
4. 6-10 hours
5. 10-12 hours
6. 12-22 hours
7. 24 hour care

How many hours of care are provided by your parents or brothers/sisters per day?

1. no
2. 0-2 hours
3. 2-4 hours
4. 4-6 hours
5. 6-10 hours
6. 10-12 hours
7. 12-22 hours
8. 24 hour care

How many hours of care are provided by healthcare providers per day?

** by caregivers we mean paid and voluntary caregivers outside the family*

1. no
2. 0-2 hours
3. 2-4 hours
4. 4-6 hours
5. 6-10 hours
6. 10-12 hours
7. 12-22 hours
8. 24 hour care

If external care providers are involved: How old are most care providers:

1. <18 years
2. 18-30 years
3. 30-50 years
4. >50 years

How many healthcare providers are involved?

a. 1-2

b. 3-4

c. >5

How did you arrange the care?

1. In kind, at home or, for example, institution/ Kokus home
2. PGB
3. PGB-PAB: ventilated; 24 hour care, living in your own place/independently (must have independent home and 24 hour care)
4. Otherwise:
5. Do not know

How is your care financed? (multiple answers possible)

1. Own finances
2. WMO (municipality)
3. WLZ (care office)
4. Health insurer (district nursing)
5. I do not know
6. Otherwise

How much time does arranging care take per week?

1. <30 minutes
2. 30 -60 minutes
3. 12 o'clock
4. 3-5 hours
5. >5 hours

Who arranges the deployment of care?

1. Myself
2. Parents (incl. foster parents/stepparents)
3. Brothers Sisters
4. Employees of an institution (think of Fokus)
5. Otherwise..

From interviews with boys and men with Duchenne, we have learned that there can be a difference between what you want and what you can do. Below are a few statements about what you would like and what you can do in practice with regard to self-care and care.

I want most of the care to be done by my parents

My parents can do most of the care

I want more care to be provided by care providers

More care can be provided by care providers

I want to choose how I want the care scheduled

I can choose how I want the care scheduled.

I would like to arrange my own care

I can arrange the care myself

I want to decide for myself what time I get up and go to bed

I can decide what time I get up and go to bed

General statements (score 0-5)

It takes me a lot of time to arrange care

I would like it if the caregivers were about the same age as me

I find it easy to find healthcare providers who are available when I need it

Receiving ventilation has made the dependency and care more intensive

Outside caregivers have less time than my parents.

How satisfied are you with your parents' care? 0 = not satisfied 5 = very satisfied

Has the care you received from your parents had an influence on your family? 0= no influence 5 = a lot of influence

How satisfied are you with the care provided by paid care providers? 0 = not satisfied 5 = very satisfied

How satisfied are you with the care provided by volunteer caregivers outside the family? 0 = not satisfied 5 = very satisfied

Are you satisfied with this division between your parents and external care providers? 0 = not satisfied 5 = very satisfied

Why/why not? …..

How important do you find the following points when it comes to your care (0 not important, 10 very important)

- Character/personality of healthcare provider 0-10
- Knowledge of healthcare worker 0-10
- Healthcare worker skills 0-10
- Moment-free/flexible division 0-10
- Fixed moments 0-10
- That you feel comfortable 0-10
- Caregiver age 0-10
- Number of caregivers 0-10
- Otherwise, namely…. 0-10

Which points have influenced the layout of your care at the moment? 0 = little 10 = a lot

- Maintaining the situation that was already there 0-10
- Availability of healthcare providers 0-10
- Time investment to arrange care 0-10
- Religion/spirituality 0-10
- Influence of parents 0-10
- Example of someone else with a care need 0-10
- Advice from care/rehabilitation center 0-10
- Advice from school 0-10
- Having ventilator support 0-10
- Living situation 0-10
- Work situation 0-10
- Future prospects with regard to living/ventilation 0-10
- Otherwise, namely ……. 0-10

Do you have any tips for someone who now has to make choices regarding care? …..

**Living situation**

How satisfied are you with your living situation? 0 = not satisfied 5 = very satisfied

Why/why not?:…

From interviews with boys and men with Duchenne, we have learned that there can be a difference between what you want and what you can do. Below are a few statements about what you would like and what you can do in practice with regard to housing

- I want to continue living with my parents

- I can continue living with my parents

- I want to live independently

- I can live independently

General statements (score 0-5)

- I am well informed about the options regarding independent living

- Living in a city offers more possibilities than living further away.

Which points influenced your choice of living situation? 0 = little 10 = a lot

1. Maintaining the situation that was already there 0-10
2. Distance to family and friends 0-10
3. Distance to social facilities 0-10
4. Distance to work 0-10
5. Facilities in the home 0-10
6. Offer of adapted homes 0-10
7. Availability of healthcare providers 0-10
8. Contact with peers 0-10
9. Financial options 0-10
10. Influence of parents 0-10
11. Example of someone else with a care need 0-10
12. Options/support from the municipality 0-10
13. Transport options 0-10
14. Otherwise, namely ……. 0-10

Have you had help in choosing a place to live? Yes No

What help did you receive in choosing a place to live? Multiple answers possible

- 1. Self-chosen
  2. Advice rehabilitation team
  3. Municipal advice
  4. Advice parents
  5. Advice peers
  6. Advice fellow sufferers
  7. Otherwise ..

Have you missed any help/advice/information regarding making choices regarding your living situation? If so, ….

1. Advice rehabilitation team
2. Municipal advice
3. Advice parents
4. Advice peers
5. Advice fellow sufferers
6. Otherwise ..

Do you have any tips for someone who now has to make choices regarding housing? …..

**Mood/ behavior**

Are you experiencing problems with your mood? Yes No

If yes which one…

How often do you suffer from this?

1. continuous
2. in periods
3. very occasionally

From interviews with boys and men with Duchenne, we have learned that there can be a difference between what you want and what you can do. Below are a few statements about what you would like and what you can do in practice with regard to your mood

I want to talk to my parents about my concerns

I can talk to my parents about my concerns

I want to talk to friends/acquaintances about my concerns

I can talk to friends/acquaintances about my concerns

I want to talk to healthcare providers about my concerns

I can talk to healthcare providers about my concerns

General statements

(never, sometimes, regularly, often, always)

I am cheerful and positive

I am sad or depressed

I get angry easily

I'm anxious

I enjoy the activities I do

If I'm not feeling well, I know where I can get help

Talking about my worries makes me feel better

I miss someone to talk to.

What factors influence your mood? 0 = little 10 = a lot

- Relationship with your parents 0-10
- Contacts with family 0-10
- Contacts with peers 0-10
- Having an (intimate) relationship 0-10
- Having the right facilities 0-10
- Having fun work or daytime activities 0-10
- Having fun hobbies 0-10
- Arranging care activities 0-10
- Availability of people to talk to 0-10
- Financial options 0-10
- Religion/spirituality 0-10
- Physical complaints 0-10
- Otherwise, namely ……. 0-10

1. Have you had professional help in processing certain emotions or mood problems?

Yes No. If yes which one…

1. If yes to one of the previous questions: How satisfied are you with the help received? 0 = not satisfied 5 = very satisfied
2. Do you have any tips for someone else with Duchenne regarding their mood? …..

**Religion/spirituality**

Are you religious? Yes No

If so, what is your faith?

Are you spiritual? Yes No

If so, what keeps you busy?

From interviews with boys and men with Duchenne, we have learned that there can be a difference between what you want and what you can do. Below are a few statements about what you would like and what you can do in practice with regard to religion and spirituality.

I have the same faith as my parents.

I want to get the most out of life.

I can get the most out of life.

I want to make my own choices about how to spend my life meaningfully.

I can make my own choices about how to spend my life meaningfully.

General statements (score 0-5)

I find my life meaningful.

I want to mean something to people around me.

I can mean something to people around me.

How satisfied are you with the role of religion/spirituality in your life? 0-5

Do you have any tips regarding religion/spirituality for others?

**Life expectancy**

Does your life expectancy play a major role in your life? Yes No

From interviews with boys and men with Duchenne, we have learned that there can be a difference between what you want and what you can do. Below are a few statements about what you would like and what you can do in practice with regard to life expectancy. (0-5)

I want to think about my future.

I can think about my future.

I want to talk to others about my life expectancy.

I can talk to others about my life expectancy.

I want to talk about death.

I can talk about death.

I have learned to deal with my decline

A limited life expectancy affects my daily activities

Can you talk to people around you about your life expectancy? Yes: multiple answers possible

1. Parents
2. Brothers Sisters
3. Friends
4. Teachers
5. Doctors/therapists from medical care
6. Otherwise …

Have you had professional help when it comes to discussing life expectancy? Yes / no, If yes, what guidance did you have/missed?

1. Parents
2. Brothers Sisters
3. Friends
4. Teachers
5. Doctors/therapists from medical care
6. Otherwise…

How satisfied are you with the help received? 0 = not satisfied 5 = very satisfied

Do you have any tips for someone else with Duchenne regarding dealing with life expectancy?

**Family life**

What does your parental family look like? Multiple answers possible

1. Two-parent family
2. Divorced parents without a new partner
3. Divorced parents with new partner
4. Well, (step) brother(s)/sister(s)
5. No (step) brother(s)/sister(s)

What are important principles within the family? Multiple answers possible:

1. Emotional connection
2. Honesty
3. Religion/spirituality/philosophy
4. Care/help each other
5. Discussing feelings towards each other
6. Otherwise, namely…

Does your mother help arrange your care? Yes No

Does your father help arrange your care? Yes No

Do you feel that your parents approach you differently than your brother(s) or sister(s)?

1. Yes
2. No
3. Bit

From interviews with boys and men with Duchenne, we have learned that there can be a difference between what you want and what you can do. Below are a few statements about what you would like and what you can do in practice with regard to the family

I want to achieve the same as my brother/sister (only for men who indicate that they have brothers/sisters).

I can achieve the same as my brother/sister

General statements (score 0-5)

I get more attention from my parents just like my brothers/sisters 0-5

Having outside caregivers limits privacy in our family.

My parents are protective.

My parents want the best for me.

My parents are my caregivers.

My parents know me best.

I need my parents if I want to do anything.

How satisfied are you with the support your parents provide in the care you need? 0 not satisfied 10 very satisfied

In your opinion, is there enough attention for brother(s)/sister(s) of boys with Duchenne? Yes No

If not, do you have any tips on how this can be improved? (open question)

Do you have a good relationship with your brother(s) and/or sister(s)? y/n

Do you have thoughts/plans for your own family? y/n?

Do you have any tips for someone else regarding their family situation?

**Social contacts**

Remark (text): Since this topic was not in your top 3 of important topics, this page has no questions. Click next to go to the next topic.

Do you have friends? Yes No

If so, how many friends do you have? ..

How many friends do you speak to online?

How many friends do you see live?

How do you know your friends? Multiple answers possible

1. Primary school
2. Secondary school
3. Work
4. Sport
5. Gaming
6. Through my parents
7. Through my brothers/sisters
8. Fellow sufferers' meetings
9. Otherwise, namely …

How much time do you spend with your friends?

1. 0-1 hours per week
2. 1-2 hours per week
3. Etc

From interviews with boys and men with Duchenne, we have learned that there can be a difference between what you want and what you can do. Below are a few statements about what you would like and what you can do in practice with regard to arranging social contacts

I want to have contact with others.

I can interact with others

I want to meet up with my friends online.

I can meet up with my friends online.

I want to have contact with fellow sufferers.

I can have contact with fellow sufferers.

General statements (score 0-5)

I make appointments myself to meet up with friends.

I decide the time to meet up with friends.

I go to friends independently

I express my emotions to friends.

My friends and I learn from each other.

How satisfied are you with your social contacts at the moment? 0 = very dissatisfied 5 = very satisfied

What influences your social contacts?

- - Parents 0-10
  - Self-confidence 0-10
  - Education 0-10
  - Work 0-10
  - Hobby 0-10
  - Family 0-10
  - Religion 0-10
  - Energy 0-10
  - Accessibility 0-10
  - Transport 0-10
  - Digital options 0-10
  - Intelligibility 0-10
  - Care needs 0-10
  - Time 0-10
  - Otherwise.. 0-10

Do you have any tips for someone else regarding social contacts?

…..

**Healthy lifestyle**

Do you drink the recommended amount (2 liters) in a day?

Do you follow a certain eating pattern? Multiple answers possible:

1. High in protein
2. High in fiber
3. Low fat
4. Low carb
5. Limited sugars
6. Vegetable
7. Otherwise …

Do you drink alcohol?

Do you use drugs?

Do you smoke cigarettes?

Do you eat >200 g of vegetables per day?

Do you eat 2 pieces of fruit a day?

Do you take nutritional supplements? Multiple answers possible:

1. Yes, Vitamin D
2. Yes, Calcium
3. Yes, Multivitamins
4. No
5. Otherwise, namely…

Are you actively working on your weight?

1. Yes lose weight
2. Yes arriving
3. No

Are you satisfied with your weight? Yes No

For me, a healthy lifestyle consists of:

- Nutrition tailored to needs.
- Drink well
- Sufficient exercise
- Otherwise, namely …

Statements: below are statements about what you want and what you want (score 0-5)

I want to have a healthy lifestyle.

I can have a healthy lifestyle.

I want to make healthy choices.

I can make healthy choices

I want to exercise enough.

I can move enough.

I want to choose my own food.

I can choose my own food.

Do you have tips for others for a healthy lifestyle? …

**Appearance**

What clothes do you wear? Multiple answers possible:

1. Clothing from (online) stores
2. Custom clothing
3. Orthopedic shoes
4. Shoes from the (online) store (e.g. sneakers)
5. ..

Statements: below are statements about what you want and what you want (score 0-5)

I want to look good.

I can look good.

I want to choose my own clothes/shoes.

I can choose my own clothes/shoes.

I want to buy my own clothes/shoes.

I can buy my own clothes/shoes.

General statements (score 0-5)

I feel more confident when I look good.

I am underestimated by those around me because I am in a wheelchair

I especially want to wear clothes that fit comfortably

I am satisfied with my appearance at the moment. 0 – 5

Which points influence your appearance/appearance:

- weight

- physical characteristics

- financial possibilities

- ease

- how I feel

- Services

- Otherwise, namely….

Do you have tips for others?

Would you like to answer questions about the other topics? Yes No

If so:

Do you have any other topics?

1. Facilities/resources
2. Transport
3. Leisure and hobbies
4. Work (paid/unpaid)
5. Education
6. Intimacy and sexuality
7. (Self) care
8. Living
9. Mood & Life Expectancy
10. Religion/spirituality
11. Family life
12. Social contacts
13. Healthy lifestyle & Appearance

Are there any other topics you would like to mention?

**(B) Survey for parents of adults with DMD**

**PART 1 General questions**

This questionnaire was completed by:

- Mother
- Father
- Stepmother
- Stepfather
- Mother and father together
- Otherwise, namely:

What is your age?

How old is your child with Duchenne?

*If 18+ Transition to adulthood?*

1. *How satisfied are you with your son's transition to adulthood? 0-10*
2. *What have been the main points that have changed? Name 3*

What is your living situation?

- Living together with partner
- Living together with partner and children
- Single
- Single with children
- Otherwise, namely,

How many children do you have?

Does your son with Duchenne live at your home?

- Yes

- No

- Partly, namely,

What kind of residential environment do you live in?

- City/region with many facilities nearby

- Outside area

Will your living situation change soon? Yes No

If so, what will change? Open question

What is your highest level of education: * according to the Dutch scholar system*

- Primary education
- Learning support education (LWOO)
- Practical education (former SVO-LOM and SVO-MLK)
- VMBO (MAVO/ULO/MULO/LAVO)
- HAVO (HBS/MMS)
- VWO
- Lower vocational education (LBO/LTS/LHNO/domestic school)
- Secondary vocational education (MBO/ROC/AOC)
- Higher vocational education (HBO/HTS/HEAO)
- University (Bachelor/Master)
- Postgraduate education

Do you work? (multiple answers possible)

- No
- Yes, paid as an employee
- Yes, paid as a self-employed person
- Yes, own company
- Yes, paid from your son's PGB
- yes, unpaid

How much time do you spend caring for your son?

1. 0 hours per day
2. 1-4 hours per day
3. 4-8 hours per day
4. 8-16 hours per day
5. 16-24 hours a day
6. Otherwise, namely.

Do you use external care providers to care for your son? Yes No

My son receives ... hours of assistance from external care providers per day (PBG/ADL assistance/home care)

What sources do you use to obtain information for your son with Duchenne? Multiple answers possible

- Instagram
- Facebook
- LinkedIn
- Websites (national help guide/ DPP/ Muscle diseases NL)
- Support grant
- Conferences and webinars
- Otherwise, namely

**Zarit Burden interview**

In this part of the questionnaire you will be presented with a number of statements that reflect how people can feel when they care for another person, in this case your son. The feelings will sometimes be recognizable, sometimes not and sometimes the statements will be confrontational.

You can always choose from five answer options, there are no good or bad answers.

|  | *1. Never* | *2.Seldom* | *3.Sometimes* | *4.Quite often* | *5.Almost always* |
| --- | --- | --- | --- | --- | --- |

| 1. Do you feel like your son is asking for more help than he needs? |
| --- |
| 1. Do you feel that because of the time you spend with your son, you don't have enough time for yourself? |
| 1. Do you feel a tension between caring for your son and your other family or work responsibilities? |
| 1. Are you ashamed of your son's behavior? |
| 1. Do you feel angry when you are with your son? |
| 1. Do you feel that your son affects your relationships with other family members or friends in a negative way? |
| 1. Are you afraid of what the future will bring for your son? |
| 1. Do you feel that your son is dependent on you? |
| 1. Do you feel tense when you are with your son? |
| 1. Do you feel that your health has suffered as a result of your involvement with your son? |
| 1. Do you feel that you have less privacy than you would like because of your son? |
| 1. Do you feel that your social life has suffered because you have to care for your son? |
| 1. Do you feel uncomfortable hosting friends because of your son? |
| 1. Do you feel that your son seems to expect you to care for him as if you were the only one he could rely on? |
| 1. Do you feel like you don't have enough money to care for your son on top of your other expenses? |
| 1. Do you feel that you will not be able to care for your son much longer? |
| 1. Do you feel like you have lost control of your own life since your son's diagnosis? |
| 1. Do you wish you could leave the care of your son to someone else? |
| 1. Do you feel unsure about what to do about your son? |
| 1. Do you feel like you should do more for your son? |
| 1. Do you feel that you could do a better job caring for your son? |
| 1. All things considered, how burdened do you feel in caring for your son? |

**Statements** :

These statements emerge from the interviews and concern the relationship between parent and son and within the family. Please indicate for the following statements to what extent this applies to you.

*1 = not applicable at all 5 = completely applicable*

**Child relationship**

I am satisfied with my relationship with my son 1-5

I give my son freedom to make his own choices 1-5

I make sure it is cozy in house 1-5

I can talk to my son about his life expectancy 1-5

I can talk to my son about limitations due to the Duchenne 1-5

I can talk to my son about intimate contacts. 1-5

My son receives the same upbringing as my other children 1-5

I encourage my son to undertake activities independently 1-5

I think it is important that my son has social contacts. 1-5

It is important to me that my son works. 1-5

I see opportunities on the labor market for my son 1-5

I see my son getting into an (intimate) relationship 1-5

I can talk well about parenting with my partner 1-5

I feel supported by my partner in the care of my son 1-5

My partner and I usually work on the same page when it comes to raising our son 1-5

I feel appreciated by my partner 1-5

A support person who can guide me through the possibilities in my son's daily life would help me. 1-5

**PART 2: Thematic questions**

**Education**

What education did your son receive? *This is according to the Dutch scholar system*

1. Regular primary education (RO)
2. Special primary education (SBO)
3. Special education (SO)
4. Special secondary education (SVO)
5. Started in regular education, switched to special education
6. Started in special education, switched to regular education
7. Otherwise, namely:

How did you experience education for your child? 0 = not appropriate 10 = very appropriate

If Special Education

Name 2 advantages of special education?

Name 2 disadvantages of special education?

If Regular Education

Name 2 advantages of regular education?

Name 2 disadvantages of regular education?

A number of topics emerged from the interviews that we would like to present to you in the following statements: (0-5)

- Regular education gives the opportunity to make friends in your own environment
- Special education fits well with the care needs
- It is possible to make a good choice before the start of education.
- It is clear what the difference is between SO and RO
- Special education provides less pressure to have to arrange things after school.

Who had the largest role in making a choice for the type of education? Multiple answers possible

1. We decided for ourselves what the best option was for our son
2. Teacher
3. My son
4. Internal supervisor
5. Ambulatory attendant
6. Healthcare providers
7. Experience of other parents of boys with Duchenne
8. Otherwise, namely:

Have you received support in making a choice for education? Yes No

What support did you receive (if yes)/miss (if no)?

1. Guidance from orthopedagogue/psychologist
2. Guidance from teacher/internal/outpatient supervisor
3. Guidance other:…
4. Information about different types of education
5. Tour of various schools
6. Participation days at different schools
7. Experience of other parents of boys with Duchenne
8. Otherwise: …

Would you make the same choice in type of education again? Yes No

If not, why not? …

If so, why? …

What would you advise parents of school-age boys with Duchenne regarding education? …

**Care**

What role do you have in your son's care?

1. I do all the care myself
2. I do all the care together with my partner
3. I arrange support in healthcare and do some of it myself
4. I arrange support for my son, but I do not have an active role in care
5. No active role
6. Otherwise …

How satisfied are you with the care given to your son? 0 = not satisfied 10 = very satisfied

How much influence do the following topics have on your satisfaction with care? Give a score between *0-10, where 0 = little influence on satisfaction and 10 = a lot of influence on satisfaction*

1. Time spent in care 0-10
2. Classification of free time 0-10
3. Availability of healthcare providers 0-10
4. Use of facilities such as lift 0-10
5. Adjustments in the home 0-10
6. Collaboration with your child 0-10
7. Safety 0-10
8. Privacy 0-10
9. Otherwise 0-10

A number of topics emerged from the interviews that we would like to present to you in the following statements:

- My role as a parent has been clouded by caring for my son 1-5
- I feel like a caregiver 1-5
- I take care of household chores 1-5
- I need support in healthcare 1-5
- If I can choose the layout of healthcare again, my share will remain the same. 1-5

How much time do you spend arranging care?

1. 1-4 hours per day
2. 4-8 hours per day
3. 8-16 hours per day
4. 16-24 hours a day
5. … hours a week
6. ... hours per month

Do you have any advice for other parents with the same care task regarding care?

**Work**

*By work we mean work other than caring for your son.*

How important is work to you? 0 = not important 10 = very important

How satisfied are you with the possibilities on the labor market when you have a son with greater care needs? 0 = not satisfied 10 = very satisfied

A number of topics emerged from the interviews that we would like to present to you in the following statements:

- I want to work. 1-5
- I can work. 1-5
- Work gives me the opportunity to have a different role. 1-5
- Working gives me the feeling that I can be myself. 1-5

Did you receive support in making the choice whether or not to continue working? Yes No

If so, what support have you had?

So what support have you missed?

Are you satisfied with your choice? 0-10

What is important to you regarding having a job? 0-10

1. Financial considerations
2. Own life
3. Different role
4. Intrinsic motivation for the work I do
5. Contacts with colleagues
6. Otherwise, namely:

Do you have any advice for other parents with the same care task regarding work? ….

**Your son's living situation**

What has the greatest influence on the choice of a place to live for your son? Make a top 3

1. Care offer
2. Transport
3. Environment
4. Financial possibilities
5. Offer of adapted homes
6. Close to family/friends
7. Facilities in the home
8. Otherwise, namely:

Are you satisfied with where your son lives? 0 = not satisfied 10 = very satisfied

A number of topics emerged from the interviews that we would like to present to you in the following statements:

(if your son lives on his own: then follow the following statements)

- I like that my son lives on his own 1-5
- I have encouraged my son to live independently 1-5
- The search for a suitable home for my son went smoothly 1-5

If no / living at home:

- I like that my son lives at home 1-5
- Independent living is desirable, but not (yet) possible 1-5
- I can help my son to live on his own 1-5
- My family needs the income from PGB 1-5

Have you received support in choosing a place to live? Yes No

What support did you have/missed when choosing a place to live? Multiple answers possible

- 1. Advice healthcare providers
  2. Options government agency (municipality)
  3. Advice fellow sufferers
  4. Advice friends/family
  5. Otherwise, namely:

Do you have any advice for other parents regarding housing? ….

**Leisure time**

How important do you think free time is? 0 = not important 10 = important

How satisfied are you with the organization of your free time? 0 = not satisfied 10 = very satisfied

Which topics influence how you organize your free time? (0-10)

1. Son's (changing) care needs
2. Labor
3. Household activities
4. Sports/other hobbies
5. Social contacts
6. Informal care for people other than your son
7. Division of tasks with partner
8. Attention to other children in the family
9. Otherwise, namely:

A number of topics emerged from the interviews that we would like to present to you in the following statements:

- I have free time in which I do not have to take into account (the care of) my son. 1-5
- I can be away for a longer period of time (overnight stays) without having to worry about my son. 1-5
- I can do the hobbies/activities that I find important. 1-5
- My family/friends give me support when necessary. 1-5
- I have peer contact with other parents with a son with Duchenne. 1-5
- I want more peer contact with other parents with a son with Duchenne. 1-5

Do you have any advice for other parents and how to fill in free time? ..

**Aids and facilities**

What is your living situation?

- Owner-occupied home
- Rental property

Are you having problems applying for benefits for your son? 0 little 10 a lot

How satisfied are you with the process of providing services for your son?

*0 not satisfied 10 very satisfied.*

A number of topics emerged from the interviews that we would like to present to you in the following statements:

- Arranging facilities goes well for me. 1-5
- I can make my own choices regarding my son's facilities. 1-5
- I want my son to arrange the facilities himself. 1-5
- My son can arrange the facilities himself. 1-5
- Arranging facilities gives me a lot of stress and takes a lot of energy. 1-5

Please rate how important you find the following topics when it comes to facilities and aids. *0 not important, 10 very important*

Own control in arranging facilities

Information offering (trade fair, website)

Know the advantages and disadvantages of facilities

Financial possibilities

Physical situation (now and in a number of years)

Advice from therapists/rehabilitation

Advice from suppliers

Knowing which organization to apply for something

Time (processing time of a request)

Knowledge and experience with therapists/suppliers regarding Duchenne

Accessibility of those involved

Otherwise, namely:

What tips do you have for other parents in the application process for facilities? ……………
